# The E50K optineurin mutation impacts autophagy-mediated degradation of TDP-43 and leads to RGC apoptosis in vivo and in vitro

**DOI:** 10.1038/s41420-021-00432-0

**Published:** 2021-03-15

**Authors:** Shiqi Zhang, Zhengbo Shao, Xinna Liu, Mingying Hou, Fang Cheng, Dawei Lei, Huiping Yuan

**Affiliations:** 1 Department of Ophthalmology, The Second Affiliated Hospital of Harbin Medical University, Harbin, China; 2 The Key Laboratory of Myocardial Ischemia, Harbin Medical University, Ministry Education, Heilongjiang Province, Harbin, China; 3 Research Institute, Second Affiliated Hospital of Harbin Medical University, Harbin, China; 4 Future Medical Laboratory, the Second Affiliated Hospital of Harbin Medical University, Harbin, China

**Keywords:** Optic nerve diseases, Retina

## Abstract

The glaucoma-associated E50K mutation in optineurin (OPTN) is known to affect autophagy and cause the apoptosis of retinal ganglion cells (RGCs), but the pathogenic mechanism remains unclear. In this study, we investigated whether the OPTN (E50K) mutation caused TDP-43 aggregation by disrupting autophagy in vivo and in vitro. OPTN (E50K) mutant mice were generated and analysed for genotype and phenotype. Adeno-associated virus type 2 vectors containing either GFP only, GFP-tagged wild-type OPTN or GFP-tagged E50K-mutated OPTN were used to transfect R28 cells. Loss of RGCs decreased retinal thickness and visual impairment were observed in OPTN (E50K) mice compared with WT mice. Moreover, overexpression of E50K OPTN induced R28 cell apoptosis. Increased p62/SQSTM1 and LC3-II levels indicated that autophagic flux was inhibited and contributed to TDP-43 aggregation in vivo and in vitro. We found that rapamycin effectively reduced the aggregation of TDP-43 in OPTN (E50K) mice and decreased the protein levels of p62/SQSTM1 and the autophagic marker LC3-II. Moreover, rapamycin increased the RGC number and visual function of E50K mice. In addition, we also observed increased cytoplasmic TDP-43 in the spinal cord and motor dysfunction in 24-month-old OPTN (E50K) mice, indicating that TDP-43 accumulation may be the common pathological mechanism of glaucoma and amyotrophic lateral sclerosis (ALS). In conclusion, the disruption of autophagy by OPTN (E50K) affected the degradation of TDP-43 and may play an important role in OPTN (E50K)-mediated glaucomatous retinal neurodegeneration.

## Introduction

Glaucoma is an important leading cause of progressive blindness worldwide. Epidemiologically, normal-tension glaucoma (NTG) is more prevalent among Asian populations, including Japanese, Korean and Chinese populations, and 21% of patients with NTG have a family history of glaucoma, indicating a genetic predisposition to the disease^1,2^. One of the genes associated with NTG is optineurin (OPTN)^3,4^, which encodes OPTN, an adaptor protein involved in a variety of cellular processes, such as signalling and autophagy^5,6^.

As an ‘autophagy receptor’, OPTN binds ubiquitin or ubiquitinated aggregates and directs them to autophagosomes^7^. It contributes to the maturation of the autophagosome^8^ and helps to degrade damaged organelles or abnormal proteins to maintain cellular homoeostasis^6^. The E50K mutation of OPTN is the most prevalent mutant form that is associated with NTG^9^; impaired autophagy has been found in E50K transgenic mice and E50K OPTN-overexpressing retinal ganglion cell (RGC)-5 cells, and this impairment was linked to apoptosis of RGCs^10–12^. Several studies have suggested that OPTN has an important role in mitophagy^9,12,13^; however, the effect of E50K on protein metabolism is rarely reported.

TAR DNA-binding protein 43 (TDP-43 or TARDBP), a known DNA- and RNA-binding protein, has a key role in mRNA processing and trafficking and microRNA biogenesis^14^. Both the ubiquitin proteasome system (UPS) and autophagy are required for TDP-43 degradation, and the clearance of cellular TDP-43 macroaggregates depends on the autophagy pathway^15^. Impairments in degradation pathways implicate failed TDP-43 clearance as a primary disease mechanism in neurodegeneration and a variety of neurodegenerative diseases, such as amyotrophic lateral sclerosis (ALS)^16–19^. To our knowledge, the link between OPTN (E50K)-mediated autophagy and TDP-43 is not clear.

To determine the effect of OPTN (E50K) on TDP-43 degradation and whether this effect has a role in glaucomatous neurodegeneration, a point mutation mouse model was developed by CRISPR/Cas9. In this study, we observed changes in TDP-43 and the level of autophagy in the OPTN (E50K) mouse model and OPTN (E50K)-overexpressing R28 cells. In addition, our study shows the effect of OPTN (E50K) on the motor system, demonstrating that TDP-43 may be the common molecular mechanism between NTG and ALS. We also examined the efficacy of the autophagic enhancer rapamycin. Our results revealed that rapamycin reduced TDP-43 accumulation in the OPTN (E50K) mouse model, and this effect was accompanied by an increase in autophagic flux and increased RGC numbers and visual function. These results indicated that TDP-43 accumulation has an important role in neurodegeneration in glaucoma and ALS.

## Results

### OPTN (E50K) mutant mice exhibit normal IOP and abnormal visual function

**Fig. 1:**
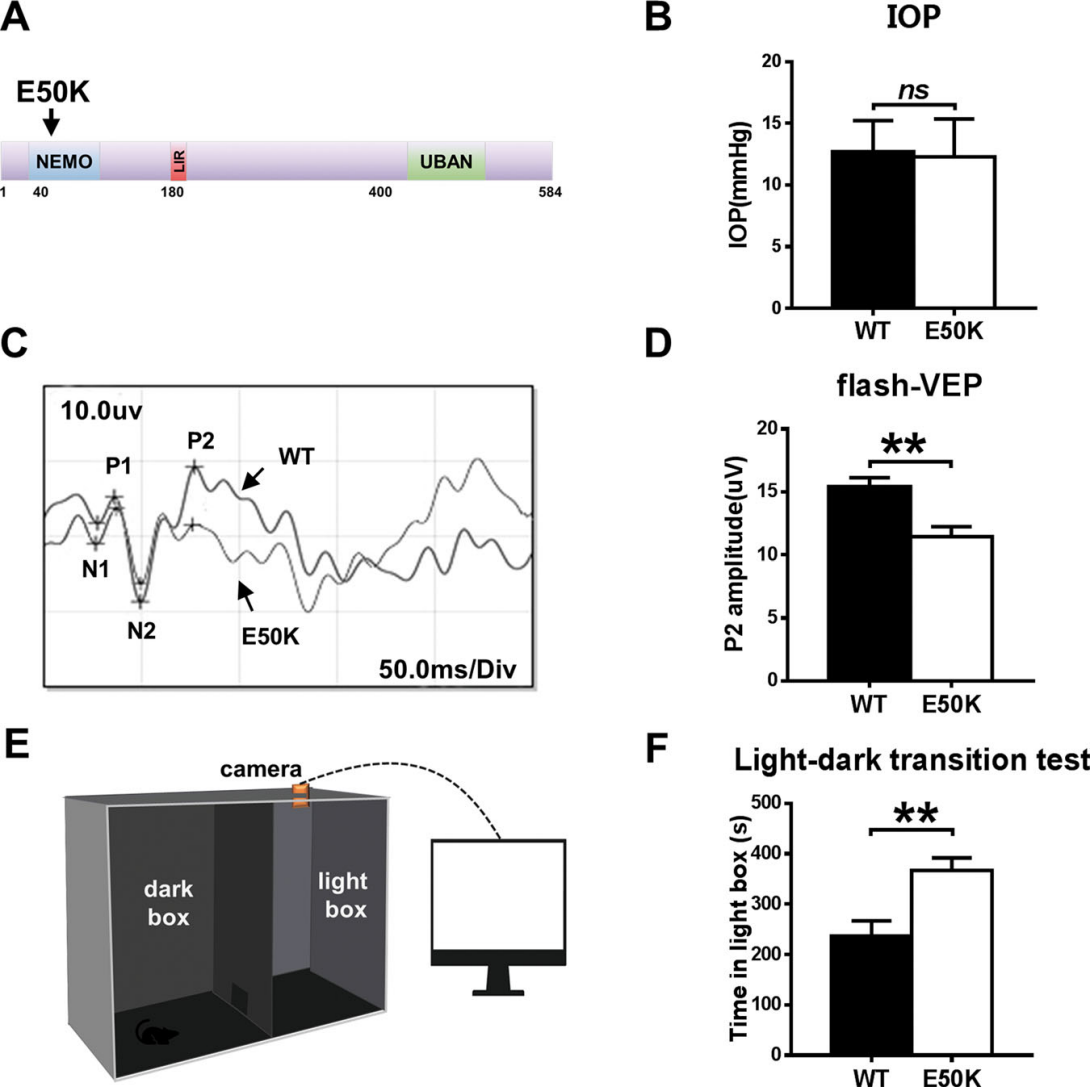
Effects of the OPTN-E50K point mutation on visual function and IOP in vivo. **A** Various domains and the E50K mutation in mouse OPTN. *NEMO* NF-kappa-B essential modulator, *LIR* LC3-interacting region, *UBD* ubiquitin-binding domain. **B** IOP values in WT and E50K mutant mouse eyes. *n* = 17. **C** Representative recorded waveforms of F-VEP and **D** decreased amplitude of P2 following visual stimulation in E50K mutant mice compared with WT mice. *n* = 48. **E**, **F** The light/dark transition test was used to evaluate visual function, and E50K mutant mice spent more time in the light box than WT mice. *n* = 7. Data are presented as the means ± SEM; *P < 0.05; **P < 0.01.

OPTN (E50K) mutant mice were generated by CRISPR/Cas9 technology. The genotypes of the mice were identified by gene sequencing, and the OPTN (E50K) mice exhibited a homozygous mutation of 148 (G > A) in the ORF region (Fig. 1A).

The OPTN (E50K) mutation is responsible for NTG^4^. We measured the IOP of wild-type and OPTN (E50K) mice. In OPTN (E50K) mice, the mean IOP was 12.06 ± 0.74 mmHg (mean ± S.E.M., *n* = 18), which was similar to the IOP measured in wild-type mice (12.71 ± 0.61 mmHg, *n* = 17) (*p* = 0.39, Fig. 1B).

However, OPTN (E50K) mice exhibit abnormal visual function without increased IOP. In the flash visual evoked potential (f-VEP) examination, the P2 amplitude measured for E50K mutant mice was 11.44 ± 0.8074 µV (mean ± S.E.M., *n* = 16), which was significantly lower than the P2 amplitude measured for wild-type mice (15.42 ± 0.7107 µV, *n* = 19) (*p* < 0.01). This result indicated that axons and the retina were damaged in the E50K mutant mice (Fig. 1C, D).

In addition, the light/dark transition test showed that the E50K mutant mice stayed in the light chamber longer than the wild-type mice (375.5 ± 39.36 s, *n* = 4 and 236.3 ± 30.79 s, *n* = 7, respectively; mean ± S.E.M., *p* < 0.05). The longer time spent in the light box suggested that the preservation of visual behaviours was impaired in the E50K mutant mice (Fig. 1E, F).

### The OPTN (E50K) mutation induces histological changes in the mouse retina

**Fig. 2:**
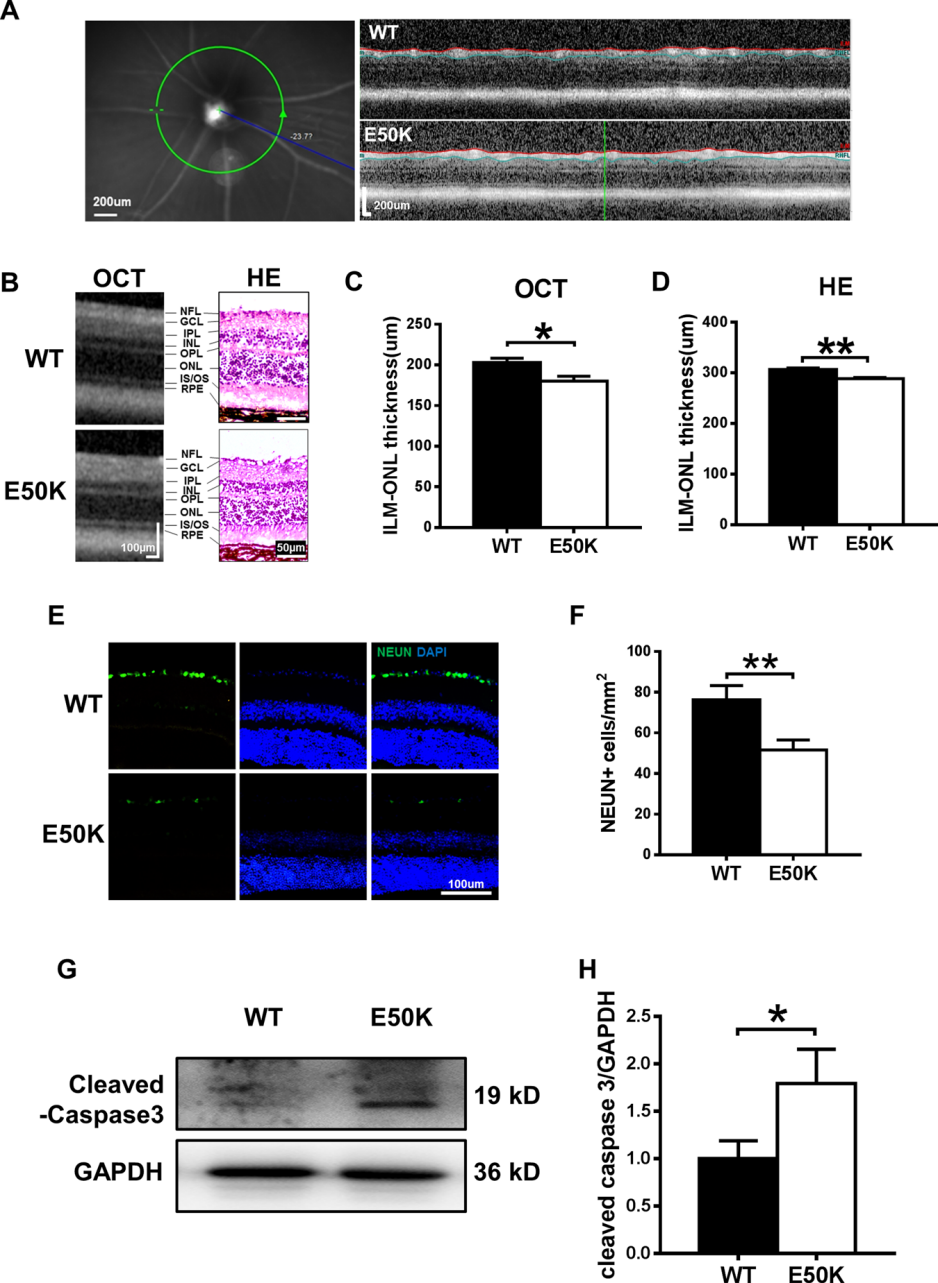
Comparison of retinal morphology and apoptosis in WT and E50K mutant mice. **A** Left: example of an SLO image of the central murine retina. Right: example of an OCT slice from WT and E50K mutant mice. **B** OCT scans and histology of retinal layers. **C**, **D** Quantification of retinal thickness measurements by OCT and histological analysis. For OCT, *n* = 10; for histology, *n* = 44/28. **E** Representative images and **F** quantification of viable RGC immunolabelling by NEUN. WT = 76.33 ± 7.000, *n* = 10; E50K = 51.67 ± 4.846, *n* = 12. **G** Caspase-3 protein expression was evaluated by western blot analysis. Representative western blot image and **H** quantification of caspase-3 protein expression in WT and E50K mutant mice. *n* = 4. Data are presented as the means ± SEM; *P < 0.05; **P < 0.01.

For the optical coherence tomography (OCT) examination, the average thickness of the retina at 3.45 mm from the centre of the optic disc was compared between the two kinds of mice (Fig. 2A). We found that the E50K mutant mice showed thinning of the retina compared with wild-type mice (180.0 ± 6.141 µm and 203.0 ± 5.143 µm, respectively; *n* = 10, mean ± S.E.M., *p* < 0.05, Fig. 2B, C). A similar result was observed in histological measurements, and it was apparent that the retinal thickness at 200 µm from the edge of the optic disc of OPTN (E50K) mice (288.5 ± 2.254 µm, *n* = 44) was significantly less than that of wild-type mice (306.5 ± 2.972 µm, *n* = 28, *p* < 0.01) (Fig. 2B, D).

As shown in Fig. 2E, F, the number of viable RGCs was significantly decreased in the OPTN (E50K) mice. In addition, the expression of cleaved caspase-3, a marker of apoptosis, was also increased in the retinas of the OPTN (E50K) mice (Fig. 2G, H).

### The OPTN (E50K) mutation induces cell apoptosis in the R28 cell line

**Fig. 3:**
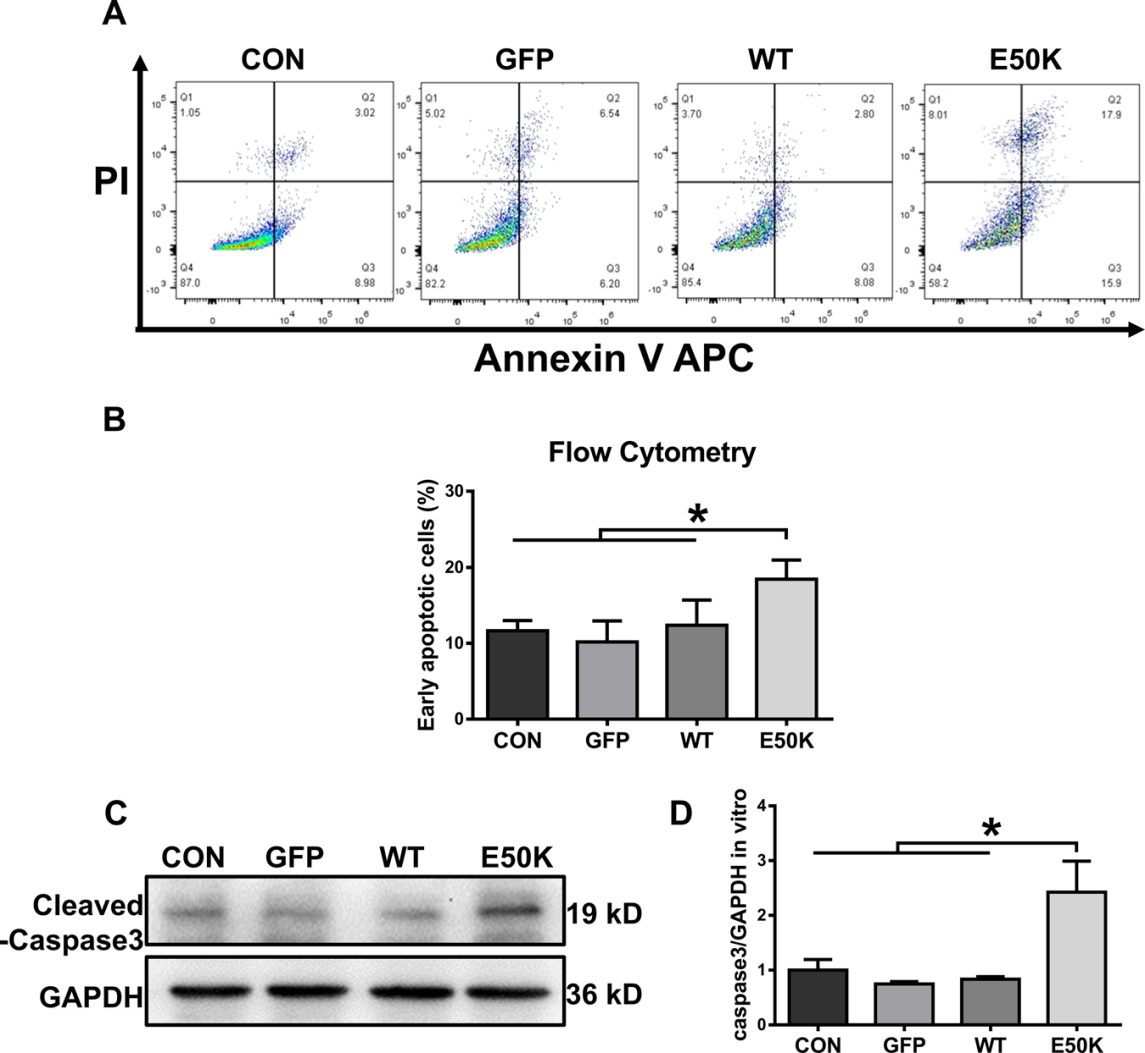
Effects of OPTN-E50K overexpression on the apoptosis of transgenic R28 cells. **A** The apoptosis rates were measured via flow cytometry and **B** quantification of early apoptotic cells 48 hours after transfection. GFP-positive cells were counted in the GFP, WT and E50K groups. *PI* propidium iodide. *n* = 3. **C** Representative western blot image and **D** quantification of cleaved caspase-3 protein expression in transfected cells. *n* = 4. Mean ± SEM. *P < 0.05.

Apoptosis in the R28 cell line was quantified by flow cytometry analysis after transfection. Annexin V-positive and PI-negative cells corresponded to early apoptotic cells. E50K OPTN-overexpressing cells (the E50K group) showed a higher early apoptotic rate than the other three groups (Fig. 3A, B, 11.66 ± 1.341, 10.19 ± 2.765, 12.39 ± 3.31 and 18.47 ± 2.517, *p* < 0.05). In addition, the protein expression level of caspase-3 was also measured in transfected cells at the same time point. Compared with the other three groups, the E50K group had increased expression of cleaved caspase-3 (Fig. 3C, D), which also suggested that the mutation could lead to increased apoptosis.

### The OPTN (E50K) mutation inhibits autophagic flux in the retina

**Fig. 4:**
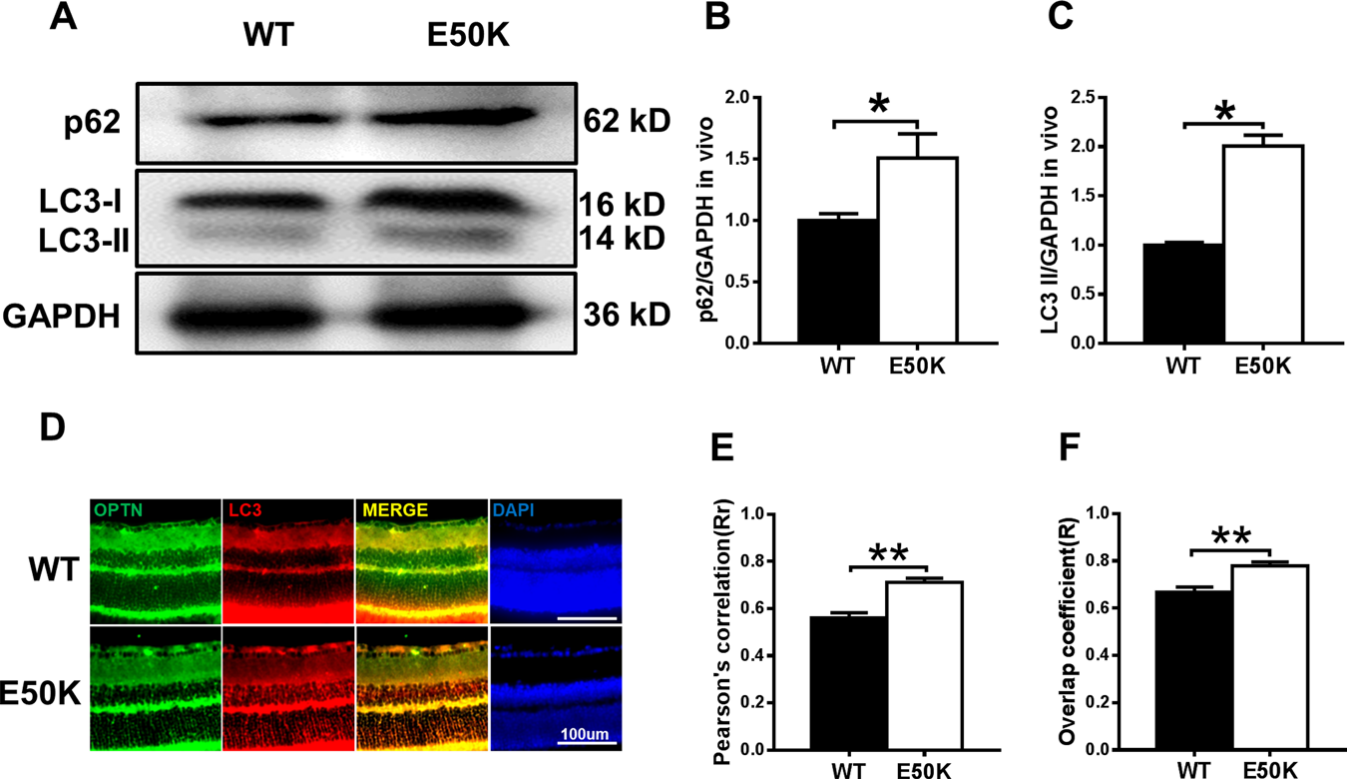
The OPTN-E50K point mutation affects autophagy and the colocalization of optineurin and LC3. **A** Western blotting showing endogenous p62/SQSTM1 and LC3 levels in retinas from WT and E50K mutant mice. **B** Quantification of p62/SQSTM1 and **C** LC3 protein expression levels. **D** Retinal sections were immunostained for optineurin and LC3, and nuclei were stained with DAPI. **E**, **F** Quantification of the percentage of OPTN-LC3 colocalization. *n* = 19/14. Mean ± SEM. *P < 0.05; **P < 0.01.

**Fig. 5:**
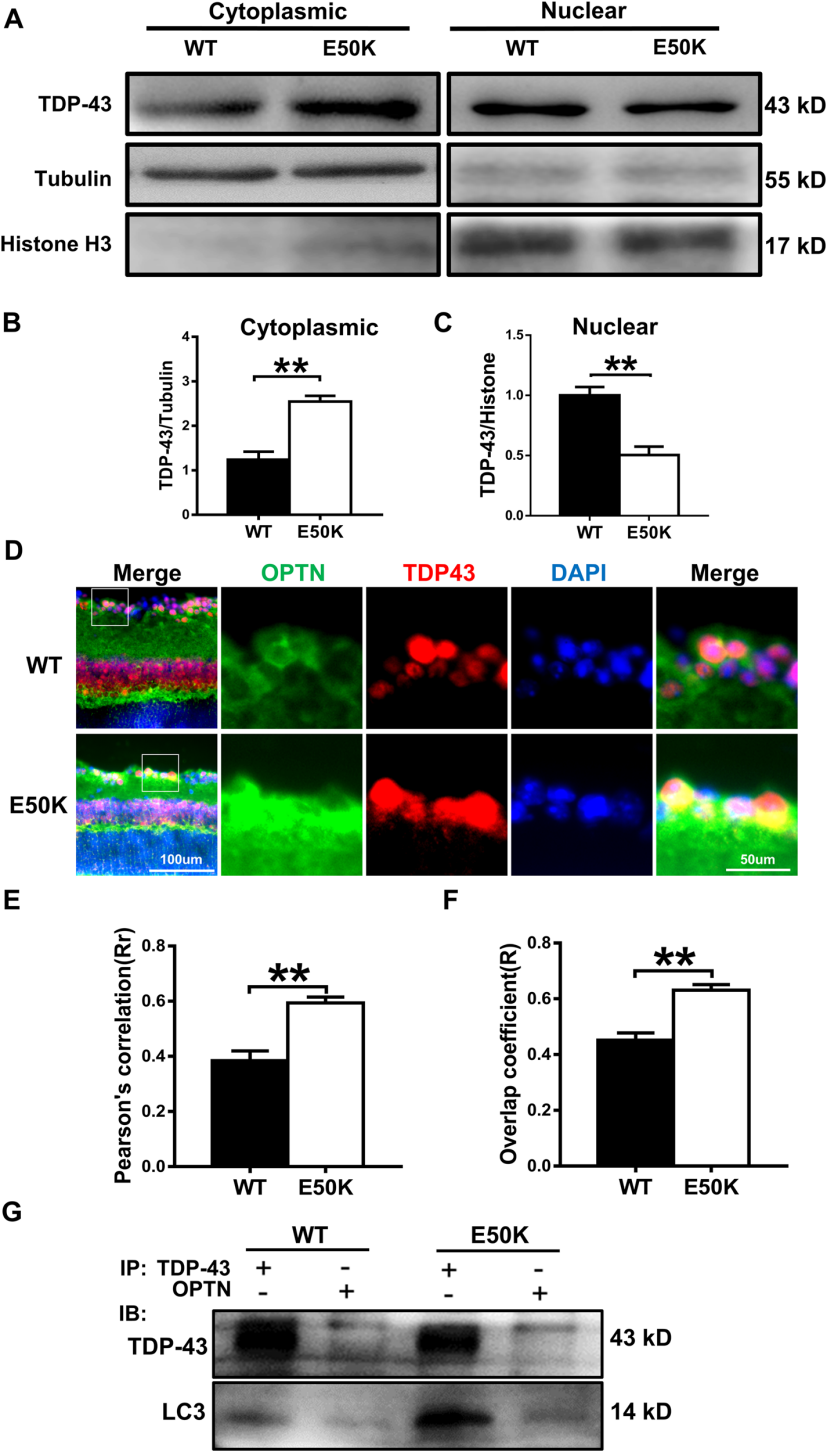
The OPTN-E50K point mutation mediates the degradation of TDP-43 via autophagy. **A** Western blot analysis of TDP-43 protein expression in the retinal cytoplasm and nucleus. β-tubulin and histone h3 were used as loading controls to validate the fractionation of cellular extracts. **B**, **C** The protein expression of TDP-43 was significantly greater in the cytoplasm and was significantly lower in the nucleus in the retinas of E50K mutant mice than in those of WT mice. *n* = 3. **D** Fluorescence analysis of the localisation of TDP-43 and optineurin in retinas of WT and E50K mutant mice. TDP-43-positive granules (red) were more colocalized with optineurin signals (green) in E50K mutant mice, and **E** and **F** both the values of Pearson’s correlation and Manders’ overlap coefficient between OPTN and TDP-43 were significantly higher in E50K mutant mice than in WT mice. *n* = 10/14. **G** Western blot analysis of immunoprecipitation assays of TDP-43 or OPTN in retinas. *P < 0.05; **P < 0.01.

To determine autophagic flux, we analysed the protein expression of LC3-II and p62/SQSTM1 in retinas. The E50K mutation increased the levels of LC3-II and p62/SQSTM1 (Fig. 4A–C, *p* < 0.05), which indicated the inhibition of autophagy. In addition, we examined the pattern of colocalization of OPTN with LC3, which is the characteristic signature of autophagic membranes^20^. The results of immunofluorescence staining revealed that both wild-type and E50K mutant OPTN colocalized with LC3 (*Rr* > 0.5, *R* > 0.5). Furthermore, a higher degree of colocalization of OPTN with LC3 was found in the OPTN (E50K) mice (Fig. 4D–F, *Rr*_WT_ = 0.560 ± 0.022, *Rr*_E50K_ = 0.712 ± 0.017, *R*_WT_ = 0.668 ± 0.021, *R*_E50K_ = 0.779 ± 0.016). The same result was also observed in coimmunoprecipitation, in which the interaction between OPTN and LC3 was enhanced in the OPTN (E50K) mice (Fig. 5F).

### The degradation of TDP-43 was affected by the E50K mutation

The effects of the E50K mutation on TDP-43 degradation were determined by analysing cytoplasmic and nuclear TDP-43 accumulation in the mouse retina. The level of cytoplasmic TDP-43 in the retinas of OPTN (E50K) mice was significantly higher than that of wild-type mice (Fig. 5A, B). Meanwhile, the level of nuclear TDP-43 was lower in OPTN (E50K) mice (Fig. 5A, C). Next, the results of immunofluorescence staining showed that TDP-43-immunoreactive aggregates tended to colocalize with E50K OPTN but not with wild-type OPTN in the retina (Fig. 5D–F, *Rr*_WT_ = 0.385 ± 0.035, *Rr*_E50K_ = 0.594 ± 0.021, *R*_WT_ = 0.452 ± 0.026, *R*_E50K_ = 0.631 ± 0.020). Interestingly, we observed a strikingly increased interaction between TDP-43 and OPTN as well as LC3 in E50K retinal tissue (Fig. 5G). This result indicated that the aggregation of TDP-43 may be associated with OPTN-mediated autophagy.

### The E50K mutation inhibits autophagic flux and affects the degradation of TDP-43 in vitro

**Fig. 6:**
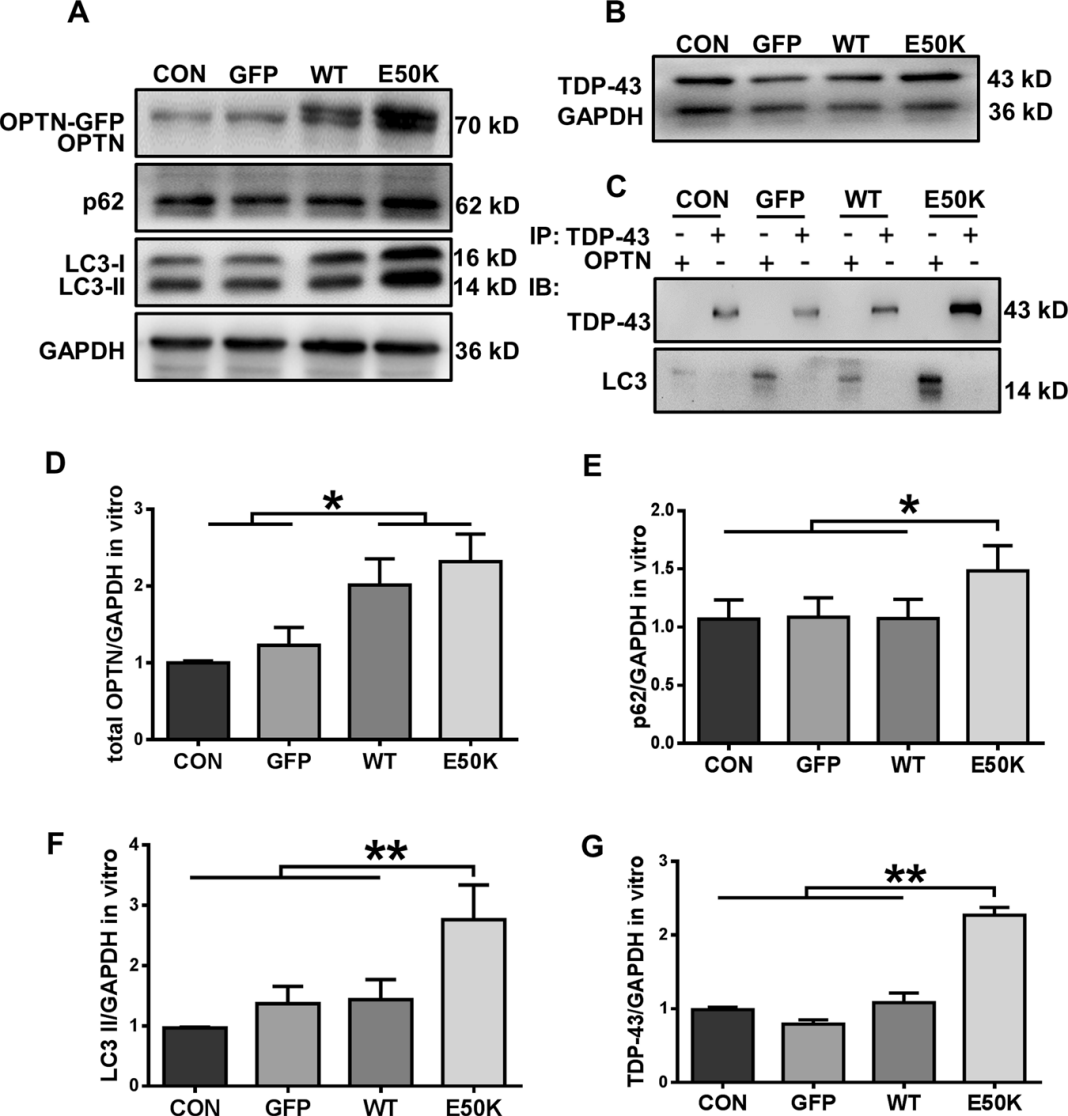
Overexpression of E50K optineurin disrupts autophagy and causes the degradation of TDP-43 in vitro. **A**, **B** OPTN, p62/SQSTM1 and LC3 protein expression and TDP-43 protein expression in the cytoplasm were analysed in blank control, AAV-GFP-, GFP-WT- and GFP-E50K-transfected R28 cells by western blotting. **C** Western blot analysis of immunoprecipitation assays of TDP-43 or OPTN in R28 cells. **D**–**F** Quantification of OPTN, p62/SQSTM1, LC3 and TDP-43 protein expression levels. *n* = 3. *P < 0.05; **P < 0.01.

In transfected R28 cells, we also detected autophagic flux and TDP-43 accumulation. Similar to the results in vivo, analysis of R28 cell protein levels showed an increase in the expression of both LC3-II and p62/SQSTM1 with a corresponding increase in TDP-43 in the E50K group, which indicated the inhibition of autophagy (Fig. 6A, B, D–G). However, the results from the in vivo and ex vivo experiments in the present study are not completely consistent. TDP-43 did not appear to interact with OPTN or LC3 in the R28 cells, whereas OPTN interacted with LC3, and their interaction was enhanced upon E50K OPTN overexpression (Fig. 6C). These results suggest that there are separate pathogenic mechanisms in vivo and in vitro.

### Rapamycin treatment increases autophagy and decreases the aggregation of TDP-43 in OPTN (E50K) mice

**Fig. 7:**
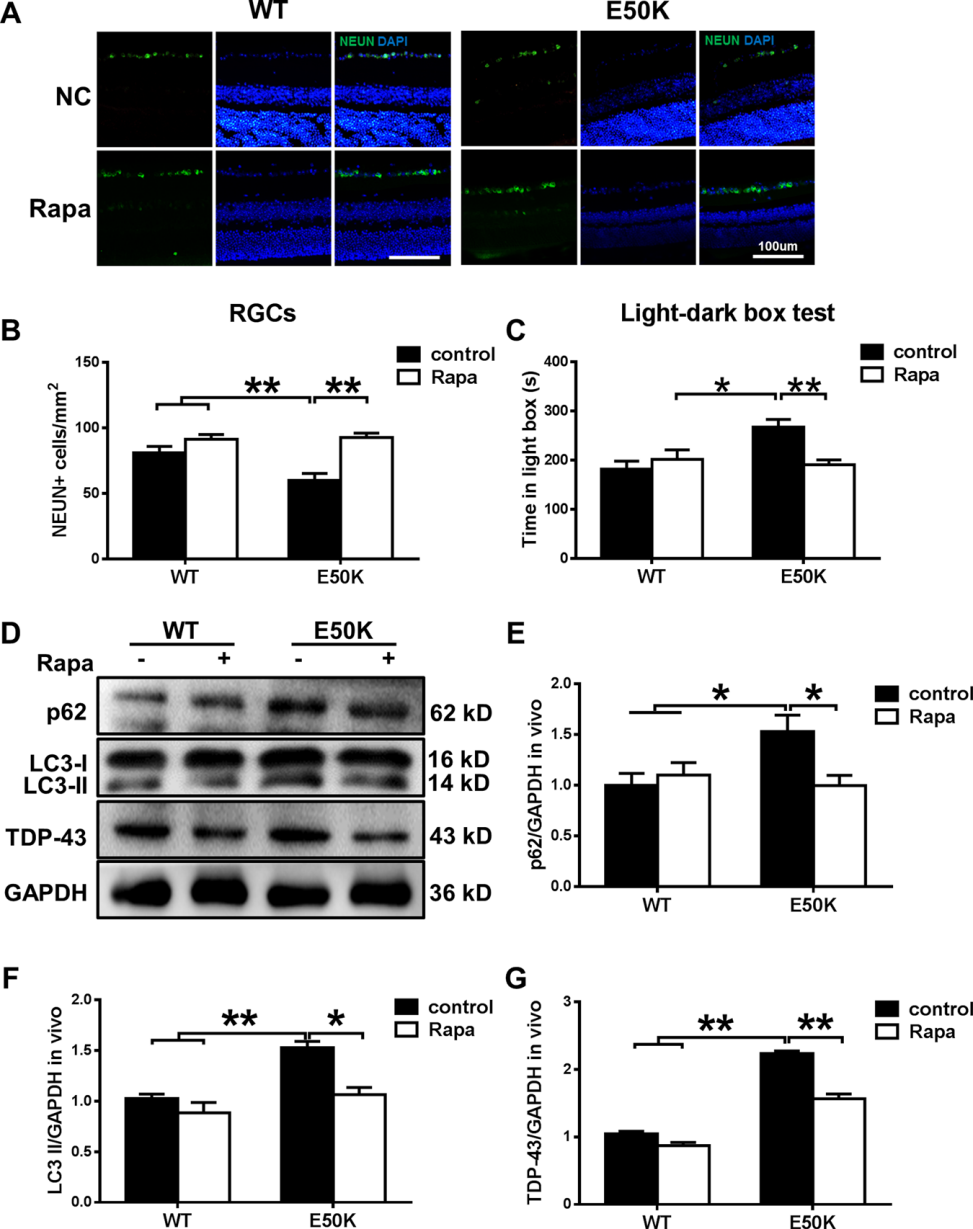
The enhanced autophagy and reduced TDP-43 levels induced by rapamycin treatment decrease E50K-induced apoptosis. **A, B** After treatment with rapamycin, the RGC number in OPTN (E50K) mice significantly increased. Representative images and quantification of viable RGC immunolabelling by NEUN are shown. *n* = 20. **C** Light/dark transition test. The time spent in the light box was recorded. Rapamycin reduced the time spent in the light box by E50K mutant mice. *n* = 8. **D** Western blot analysis of p62/SQSTM1, LC3 and TDP-43 protein expression in the retinas of mice and **E**, **F** quantification of protein expression levels. **G** The expression of TDP-43 protein was significantly decreased after rapamycin treatment. *n* = 3. *P < 0.05; **P < 0.01.

To elucidate the effects of rapamycin, WT and OPTN (E50K) mice were treated as described in the methods. After 5 weeks, the RGC number of the E50K-rapamycin group was significantly increased (Fig. 7A, B). Moreover, the light/dark transition test showed that rapamycin treatment improved the visual function of OPTN (E50K) mice. We also analysed endogenous LC3-II and p62/SQSTM1 modification in mouse retinas. The rapamycin-treated E50K mutant mice demonstrated a decrease in LC3-II and p62/SQSTM1 expression levels (Fig. 7D–F). Furthermore, the aggregation of TDP-43 was significantly decreased in E50K mutant mice after rapamycin treatment (Fig. 7D, G). Collectively, these results indicate that the degradation of TDP-43 was associated with autophagy and RGC viability and function in the mutant mice.

## Discussion

In the present study, we investigated the pathogenic mechanisms of E50K-induced RGC apoptosis in a 16-month-old CRISPR/Cas9-mediated mouse model and an OPTN (E50K)-overexpressing R28 cell model. We found a strong association between increased autophagy-mediated pathologic TDP-43 levels and increased RGC apoptosis in the E50K mutant mice. Rapamycin showed a visual protective effect by reducing RGC apoptosis, which may be related to upregulation of autophagy and decreased levels of TDP-43. Our results are helpful for a better understanding of the underlying mechanism of NTG caused by OPTN (E50K) and targeted treatment of NTG.

In the clinic, retinal thickness changes can be observed in POAG and NTG patients. In addition, it has previously been reported that overexpression of E50K mutant OPTN in transgenic mice induces apoptosis of RGCs, leads to progressive retinal degeneration after 16 months^3,21,22^ and shows a mild glaucoma phenotype^23^. However, because of endogenous OPTN or the overexpression of E50K OPTN, it remains unclear whether the mechanisms of this induction are comparable to the situation seen in glaucoma. To avoid this problem, we used a point mutation mouse model to study the glaucoma phenotype of humans.

Similar to previous studies, we showed that OPTN (E50K) mutant mice exhibited thinning of the retinal layers, loss of RGCs and functional visual impairment without higher IOP (Figs. 1 and 2). These findings indicated that the E50K point mutation induced toxic effects such as apoptosis so that the mutant mice showed an NTG-like phenotype. Based on these results, CRISPR/Cas9 produced an ideal mouse model for evaluating the pathogenic mechanism of NTG.

Without higher IOP, neurodegeneration possibly occurs through a different disease mechanism. Therefore, we next studied the possible underlying mechanism in OPTN-E50K point mutant mice. OPTN belongs to a group of ubiquitin-binding autophagy receptors. It is widely thought to link ubiquitinated cargo through ubiquitin-binding domains to autophagosomal membranes by binding to Atg8 family proteins^24^; thus, abnormal function of OPTN is closely related to impaired autophagy. Autophagy is a crucial mechanism that helps maintain TDP-43 homoeostasis by removing larger aggregates through lysosomal degradation. It has been reported that overexpression of E50K OPTN induces a decrease in autophagosome formation to inhibit autophagic flux in RGC-5 cells^10^. Moreover, autophagy impairment also occurs in adeno-associated virus type 2 (AAV2)-OPTN_E50K_-transduced eyes of rats^11^. OPTN-E50K would be expected to lead to an inability to remove aggregates; thus, we hypothesised that impaired autophagic flux may result in the formation of TDP-43 aggregates in the OPTN-E50K mutant mouse.

To test this conjecture, we measured the level of autophagic flux, which is usually monitored by detecting endogenous LC3-II modification and p62/SQSTM1 aggregation. Because increased LC3-II may be caused by autophagy activation or the failure of autophagic lysosome clearance^25,26^, p62/SQSTM1 was used to help determine the level of autophagy. The accumulation of p62/SQSTM1, a marker of autophagy, is observed in response to autophagy inhibition^27,28^. Therefore, increased levels of p62/SQSTM1 and LC3-II indicate the inhibition of the late stage of degradation, which is usually regarded as the inhibition of autophagic flux. In our results, increased LC3-II and P62/SQSTM1 were observed, confirming the impairment of autophagy in E50K mutant mouse retinas.

During the process of autophagy, OPTN recognises protein aggregates to regulate the clearance of abnormal proteins, helping to maintain cellular homoeostasis^29^. TDP-43 is aggregation-prone, and its accumulation in the cytoplasm correlates with toxicity^14^. Clearance of larger TDP-43 aggregates occurs via the autophagy pathway^14,30^. Thus, autophagy-mediated protein clearance has an important role in the balance of TDP-43 synthesis. Moreover, Yamashita et al.^31^ and Maruyama et al.^32^ presented evidence that OPTN is associated with TDP-43 and that TDP-43-positive inclusions showed positive immunolabelling with anti-OPTN antibodies in the muscles of patients with sporadic inclusion body myositis and sporadic ALS.

In our study, we observed a significantly higher level of TDP-43 in the cytoplasm of retinas from OPTN (E50K) mice than in those from wild-type mice, accompanied by a reduction in nuclear expression (Fig. 5A–C). Furthermore, both fluorescence microscopy and coimmunoprecipitation showed that OPTN exhibited a degree of increased interaction with TDP-43 in OPTN (E50K) mice. In addition, an increased interaction between LC3 and OPTN or TDP-43 was observed in the retinas of mutant mice. These results revealed that OPTN (E50K) caused TDP-43 to accumulate more frequently in the cytoplasm and deplete in the nucleus of retinas, and these changes were related to E50K-mediated autophagy because of the altered interactions. To our knowledge, this is the first report of abnormal TDP-43 accumulation in the retinas of OPTN (E50K) mice.

The formation of toxic protein aggregates is associated with several neurodegenerative diseases^33^. TDP-43 aggregation, which is referred to as TDP-43 proteinopathy, is usually identified in ALS^34,35^. Moreover, some mutations in OPTN, such as the E478G and D477N mutations, have been found to cause ALS, suggesting that there is a common pathological mechanism between these two diseases^36^. Moreover, it has also been reported that retinal neuron loss is preceded by the mislocalization of TDP-43 in Grn-KO mice^37^ and that increased cytoplasmic TDP-43 induces retinal degeneration, including thinning of the retina, in a Drosophila ALS model^38,39^.

However, no OPTN mutation has been reported to cause either glaucoma or ALS, and no E50K-linked case of ALS has been reported. Although the E50K mutation leads to abnormalities in TDP-43 in the retina, increased TDP-43 expression does not always result in TDP-43 pathology^40,41^, so we cannot determine the effect of E50K on the motor system. To answer this question, rotarod tests were used to assess whether the knock-in mice would develop ALS-like phenotypes. Unlike visual function, the knock-in mice appeared indistinguishable from age-matched wild-type mice until 18 months of age (Figure S1). Surprisingly, 24-month-old heterozygous OPTN (E50K) mice exhibited significant motor dysfunction. Moreover, the amount of cytoplasmic TDP-43 was increased significantly in the spinal cord of 24-month-old but not 16-month-old knock-in mice (Figure S2). Intriguingly, it seems that E50K-induced TDP-43 abnormalities and dysfunction earlier in retinas than in the spinal cord. The reason for the differences between the two systems remains to be discovered. The present results further indicate that TDP-43 abnormalities may be the common mechanism of NTG and ALS. Therefore, a mutation in an ALS-associated mutation knock-in model, such as E478G, may help clarify the common and different pathological mechanisms of TDP-43 in these two diseases.

Moreover, recent evidence identified RGC-5 cells as the 661 W photoreceptor cell line and indicated that RGC-5 cells are not of RGC origin, so in the current study, we used the rat retina R28 cell line, which has been characterised and used in a variety of in vitro studies of retinal cell behaviour^42^ to investigate the effect of E50K. Similar to the in vivo results, overexpression of OPTN-E50K also induced apoptosis, suppressed autophagy and affected TDP-43 degradation in the R28 cell line. However, coimmunoprecipitation analysis in transfected R28 cells yielded inconsistent results. TDP-43 did not appear to interact with OPTN or LC3 in R28 cells, whereas OPTN interacted with LC3, and their interaction was enhanced upon E50K OPTN overexpression. The possible cause may be associated with the differences between knock-in (in vivo) and overexpression (in vitro). First, in contrast to the knock-in model, E50K OPTN-overexpressing cells express endogenous OPTN, which may affect the results. Furthermore, it is thought that the impairment of proteasomal degradation leads to the initial formation of TDP-43 aggregates, and then impairment of autophagy prevents the removal of aggregated TDP-43^15^. Ubiquitin proteasome pathway (UPP) function was also reduced by overexpression of E50K OPTN^11^, so we concluded that the UPP was affected first, leading to the accumulation of TDP-43 owing to the limited transfection time. Although the autophagy pathway was also inhibited, it was not the main factor contributing to the changes in TDP-43 in vitro. Moreover, overexpression of OPTN can also cause an imbalance in cell homoeostasis. Therefore, it is difficult to evaluate the pathological effects of OPTN (E50K) compared with those of wild-type OPTN without appropriate control of their levels of overexpression in transfected cells. This result indicated the importance of the knock-in model in investigating the pathogenesis of the E50K mutation in OPTN. However, validation of this hypothesis will require further investigation.

To confirm the role of OPTN-mediated autophagy in TDP-43 degradation, we adopted the autophagy activator rapamycin. Rapamycin has been reported to compromise proteasome function and activate the autophagic process in E50K-transduced rat eyes and was effective in rescuing adverse OPTN phenotypes^11^. In our study, we found that rapamycin was effective in reducing the aggregation of TDP-43 in our mouse model and induced decreases in the p62/SQSTM1 protein and the autophagic marker LC3-II (Fig. 7). These results indicate that the burden of TDP-43 accumulation will be diminished by enhancing autophagic flux. Moreover, rapamycin increased the RGC number and visual function of OPTN (E50K) mice. This result indicates that the use of rapamycin to reduce TDP-43 could suppress E50K-mediated apoptosis. All the above-mentioned results support our hypothesis that OPTN-E50K inhibits autophagy, leading to TDP-43 aggregation and RGC apoptosis.

In conclusion, our results showed that the glaucoma-associated E50K OPTN mutation induced RGC reduction and visual impairment in vivo and cell apoptosis in vitro. The disruption of autophagy by OPTN-E50K affected the degradation of TDP-43 and may play an important role in E50K-mediated glaucomatous neurodegeneration.

## Materials and methods

### Development of OPTN (E50K) point mutation mice

The experiments in mice were approved by the Institutional Animal Care and Use Committee of Harbin Medical University and performed following the Statement for the Use of Animals in Ophthalmic and Vision Research by the Association for Research in Vision and Ophthalmology. OPTN containing the E50K mutation was created through homologous recombination-mediated knock-in using the CRISPR/Cas9 system. In brief, the donor vector was constructed containing two homologous arms and a point mutation region. Cas9 mRNA, gRNA and donor vector were microinjected into fertilised eggs from C57BL/6 mice. To avoid CRISPR off-target events, F0 lines were outcrossed to F2. Heterozygous F2 animals were intercrossed to generate mutant and wild-type mice. We examined mice after the F3 generation, and the genotypes of the mice were confirmed by sequencing of PCR fragments (896 bp) in the point mutation target region amplified from genomic DNA isolated from blood using the following primers: forward, AGCCGGGCAGCGTTAACTGGATG; reverse, CTCACTCTGGGGCCCTGTTCATTC. The mice were maintained on a 12-hour light/dark circle. Sixteen-month-old mice were used in our study, and the researchers were not blinded to the grouping of the mice during experiments or analysis.

### Cell culture and transfection

The R28 retinal precursor cell line was cultured in low-glucose Dulbecco’s modified Eagle’s medium with 10% calf serum, 100 U/ml penicillin and 100 mg/ml streptomycin in a 37 °C humidified atmosphere containing 5% CO_2_. Cells were transfected with AAV2-EGFP, AAV2-OPTN_WT_-EGFP and AAV2-OPTN_E50K_-EGFP (Hanbio, Shanghai, China), which were constructed by inserting the mouse wild-type or E50K OPTN gene according to the manufacturer’s instructions. Cells were collected through trypsin digestion 48 hours after transfection.

### Intraocular pressure measurement

A rebound tonometer (Tonolab, iCare Finland Oy, Helsinki, Finland) was used to measure the intraocular pressure (IOP) of mice according to the manufacturer’s directions. Seven readings were taken per eye immediately after the mice were fully anaesthetised. The average values of WT and OPTN (E50K) mice were compared and analysed by Student’s *t* test.

### Visual function detection

F-VEP and light/dark transition tests were used to detect the visual function of mice as previously described^43^. In brief, the active electrode, reference electrode and ground electrode were inserted under the skin of the occiput, mandible and upper limb of mice to perform f-VEP. The waveform was recorded three times for each eye, and the average P2 amplitude was analysed. A light/dark transition test was conducted as described previously^44^. After 2 hours of dark adaptation, the mouse was put into the dark box (Fig. 1E), and data were collected for 10 min to evaluate visual function by determining the time spent in the light box.

### Retinal thickness measurements

We measured the thickness of the inner five layers of the retina by OCT (Heidelberg, Germany) and histology. Mice were placed on a platform after anaesthesia and pupil dilation and adjusted to ensure that the incident beam was perpendicular to the central cornea and passed through the pupil. The retinal thickness of mice at a circle with a diameter of 3.45 mm (system setting) using the optic disc as the centre was measured by OCT with a 25D lens (50744, Heidelberg, Germany)^45^. After the measurement of live animals, the mice were anaesthetised and cardiac perfused with 4% paraformaldehyde in phosphate buffer. The eyeballs were removed and fixed in 4% paraformaldehyde overnight at 4 °C. After fixation, the posterior segment of the eyeball was dehydrated in 25% sucrose overnight and embedded in OCT compound (Catalogue no. 4583; Sakura Finetek, Tokyo, Japan). Transverse sections (6 µm thick) through the optic disc of the eye were made for Hematoxylin and Eosin staining. All measurements were performed 2 mm away from the optic disc edge.

### Immunofluorescence

Antigen retrieval was performed using a high-pressure method in 0.01 M sodium citrate-hydrochloric acid buffer (pH = 6.0) on tissue sections for 2 min, followed by immersion in antigen retrieval solution (C1035, Solarbio Science & Technology, China) for 5 min and blocking with 5% goat serum containing 0.3% Triton X-100 for 1 hour at room temperature. Then, the sections were incubated with the following antibodies at 4 °C overnight: rabbit anti-OPTN (1:200, 10837-1-AP, Proteintech Group), mouse anti-TDP-43 (1:200, sc-376311, Santa Cruz), mouse anti-LC3β (1:200, sc-271625, Santa Cruz) and rabbit anti-NEUN (1:50, ab177487, Abcam). After the primary antibodies, the sections were incubated with fluorescein isothiocyanate-AffiniPure goat anti-rabbit IgG (1:250, 111-095-003, Jackson) and Red-X-AffiniPure goat anti-mouse IgG (1:250, 115-295-003, Jackson) antibodies, and the cell nuclei were counterstained with DAPI (C1005; Beyotime Co., Shanghai, China) at room temperature for 3 min and placed in anti-fade fluorescence mounting medium. All images were acquired by fluorescence microscopy (Olympus Corporation, Tokyo, Japan), and Pearson’s correlation coefficient (*Rr*) and Manders’ overlap coefficient (*R*) were calculated by Image-Pro Plus software to analyse the colocalization. NeuN-positive cells were counted by ImageJ from 24 images. Four mice from each group were collected.

### Western blot

After removing the anterior portion of the eye and the lens, retinas were lysed in radioimmunoprecipitation lysis buffer (P0013B, Beyotime Co.) to extract proteins. For R28 cells, proteins were extracted in the same way. Cytoplasmic proteins were isolated by a Nuclear and Cytoplasmic Extraction Kit (CW0199, CWBio) according to the manufacturer’s instructions. Twenty micrograms of pooled retinal protein (*n* = 6 retinas/group) or cellular protein were resolved by sodium dodecyl sulphate polyacrylamide gel electrophoresis and transferred to polyvinylidene fluoride membranes. Western blot analysis was performed as described^44^. The primary antibodies included rabbit anti-OPTN (1:1000, 10837-1-AP, Proteintech Group), p62/SQSTM1 (1:1000, 18420-1-AP, Proteintech Group), LC3A/B (1:1000, #4108, CST), caspase-3 (1:1000, 19677-1-AP, Proteintech Group), TDP-43 (1:1000, 10782-2-AP, Proteintech Group), GAPDH (1:1000, 10494-1-AP, Proteintech Group), histone H3 (1:1000, 14269 S, CST) and mouse anti-β-tubulin (1:2000, bsm-33034M, Bioss) antibodies. The immunoblots were quantitated by using ImageJ software.

### Measurement of cell apoptosis

Apoptosis was measured using an APC Annexin V Apoptosis Detection Kit with PI (640932, Biolegend) according to the manufacturer’s instructions. In brief, after transfection, cells were collected, washed, resuspended in 100 μl of annexin V binding buffer and then stained with 5 μl of annexin V and 10 μl of PI in the dark for 15 min at room temperature. The percentage of nonviable apoptotic cells among the transfected cells was evaluated by flow cytometry (BD FACSCanto II Flow Cytometer; BD Bioscience).

### Treatment with rapamycin

To examine the effects of rapamycin, 15-month-old WT and OPTN (E50K) mice received 3 mg/kg rapamycin via IP injection three times per week for 5 weeks^11,46^. The half-life of rapamycin is ~3 days. After treatment, we analysed the visual function of the mice by the light/dark transition test. Later, the mice were killed, and we counted the number of RGCs in the retina. In addition, we tested the protein levels of p62/SQSTM1, LC3 and TDP-43 in the retina by western blotting.

## Supplementary information

Figure S1

Figure S2

Figure S3
