# Supplementary figures and images for: The E50K optineurin mutation impacts autophagy-mediated degradation of TDP-43 and leads to RGC apoptosis in vivo and in vitro
Source: Cell Death Discov. 2021 Mar 15;7:49. doi: 10.1038/s41420-021-00432-0 (PMC7960725; doi:10.1038/s41420-021-00432-0)

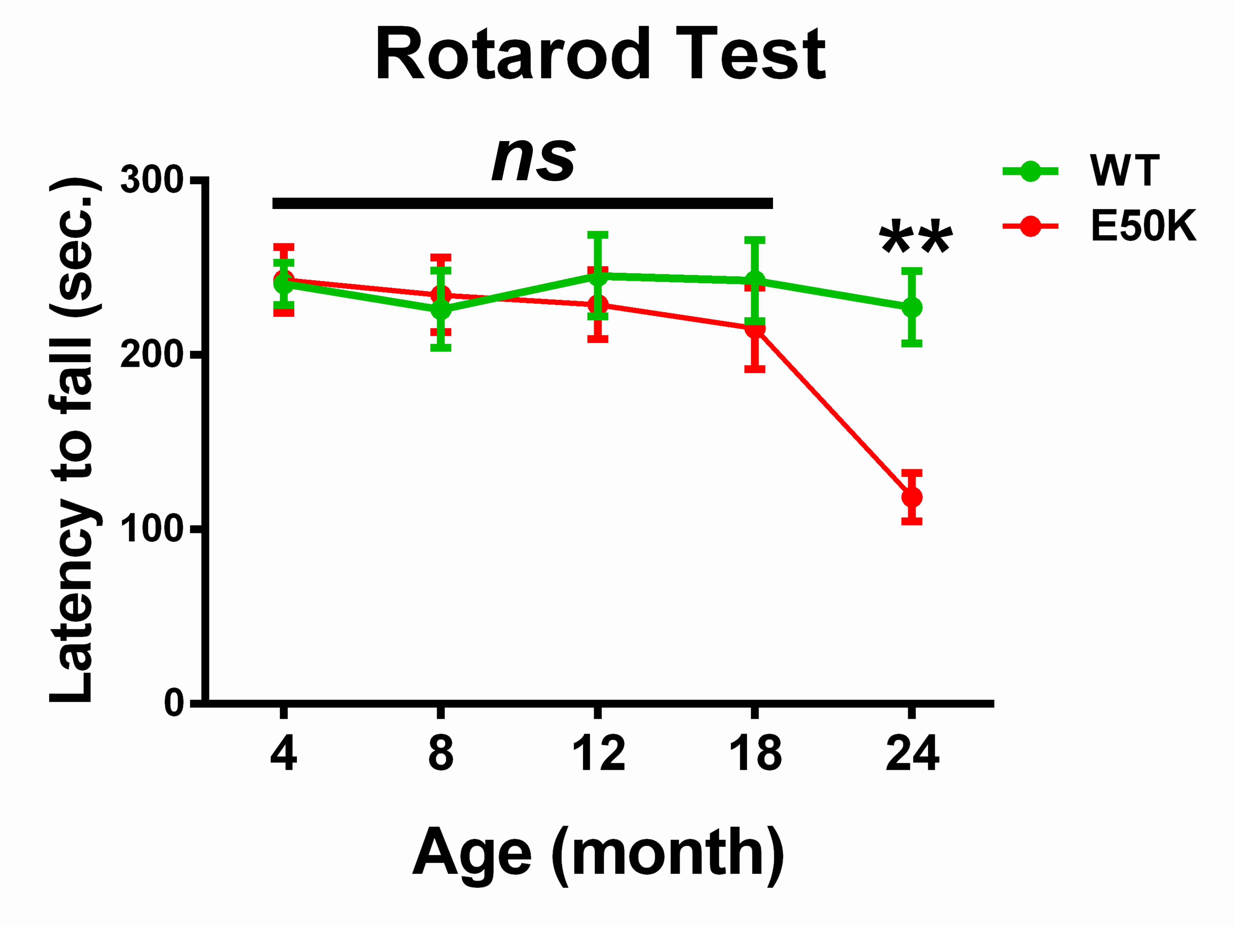

Supplement: Supplementary file 1 — Figure S1 [file 41420_2021_432_MOESM1_ESM.tif]

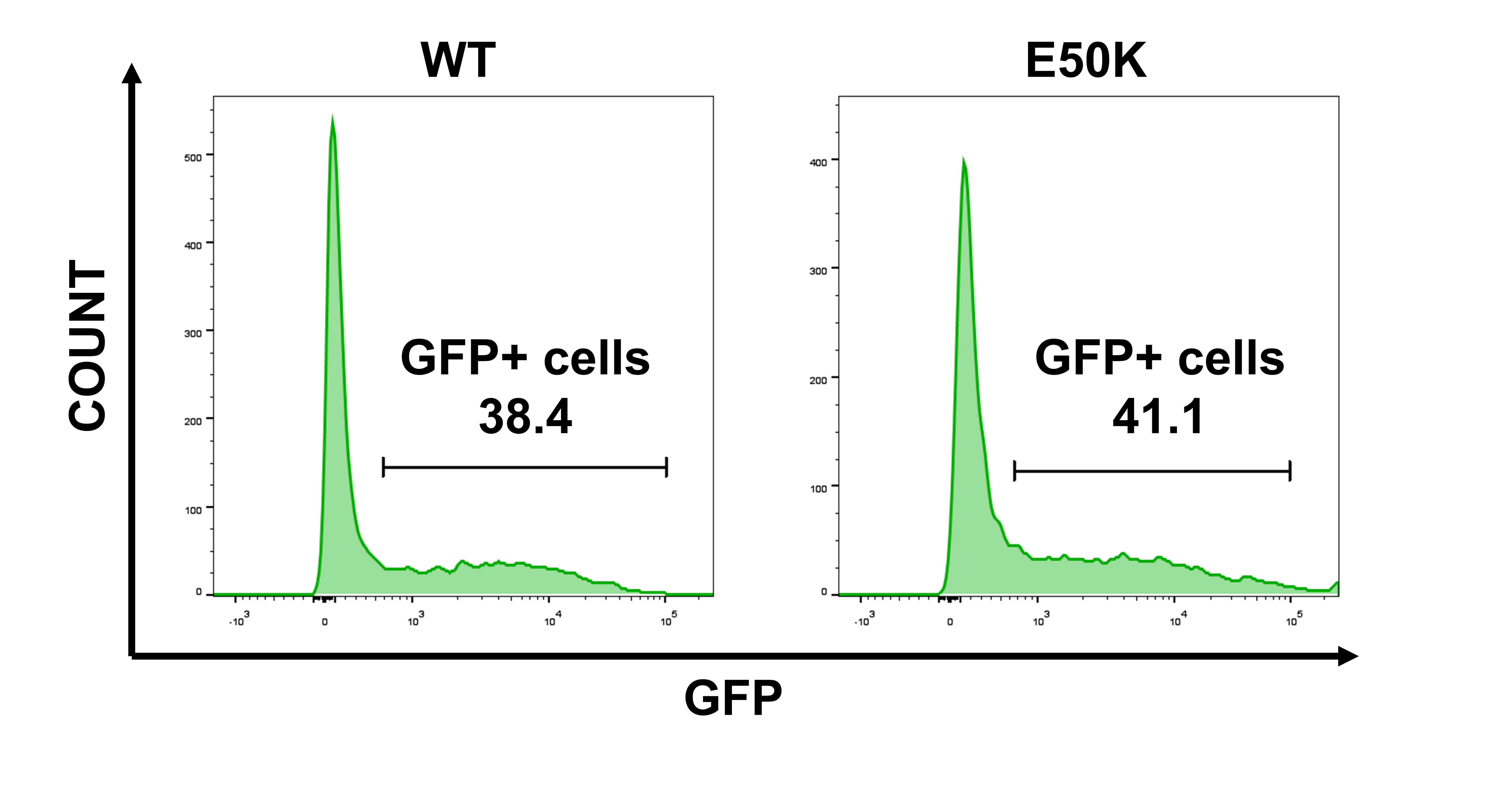

Supplement: Supplementary file 3 — Figure S3 [file 41420_2021_432_MOESM3_ESM.tif]
